# Back to the Future: Achieving Health Equity Through Health Informatics and Digital Health

**DOI:** 10.2196/14512

**Published:** 2020-01-14

**Authors:** LaPrincess C Brewer, Karen L Fortuna, Clarence Jones, Robert Walker, Sharonne N Hayes, Christi A Patten, Lisa A Cooper

**Affiliations:** 1 Department of Cardiovascular Medicine Mayo Clinic College of Medicine Rochester, MN United States; 2 Dartmouth College Lebanon, NH United States; 3 Hue-Man Partnership Minneapolis, MN United States; 4 Massachusetts Department of Mental Health Boston, MA United States; 5 Department of Psychiatry and Psychology, Mayo Clinic College of Medicine Rochester, MN United States; 6 Division of General Internal Medicine, Department of Medicine, Johns Hopkins University School of Medicine Baltimore, MD United States

**Keywords:** health informatics, digital health, mobile health, eHealth, community-based participatory research, health equity

## Abstract

The rapid proliferation of health informatics and digital health innovations has revolutionized clinical and research practices. There is no doubt that these fields will continue to have accelerated growth and a substantial impact on population health. However, there are legitimate concerns about how these promising technological advances can lead to unintended consequences such as perpetuating health and health care disparities for underresourced populations. To mitigate this potential pitfall, it is imperative for the health informatics and digital health scientific communities to understand the challenges faced by disadvantaged groups, including racial and ethnic minorities, which hinder their achievement of ideal health. This paper presents illustrative exemplars as case studies of contextually tailored, sociotechnical mobile health interventions designed with community members to address health inequities using community-engaged research approaches. We strongly encourage researchers and innovators to integrate community engagement into the development of data-driven, modernized solutions for every sector of society to truly achieve health equity for all.

## Introduction

There has been recent growth in the use of high-tech health devices such as exercise trackers, heart rate monitors, and other devices. There has also been an explosion of new ways of working with health information and health care providers (doctors, nurses, community health workers, etc), including video doctor visits, text message reminders to take medicine or exercise, and other ways for people to get their health information when and how they want and need it. These devices and the way they are used are known as health informatics and digital health. Their use will continue to grow and impact the health of many people. But, there are real concerns about how these technologies may lead to bad effects. For example, technology may cause differences in health for groups of people without many resources. It is important that people and companies who develop these new technologies understand the challenges faced by disadvantaged groups. These challenges prevent community members from being as healthy as possible. This paper gives examples of health programs using technology created *with* community members to help them improve their health. These programs are based on where people live, work, play, and pray. We believe that researchers and developers should work together with communities to build modern tools to make everyone healthier.

## Digital Disadvantage for the Disadvantaged

“The world of Pokémon GO is all around you…!” This was the seemingly all-embracing community experience promised by the highly popular augmented reality game, Pokémon GO (Niantic, Inc), one of the most frequently used mobile apps worldwide [1,2]. The app-based game was centered on the premise of incentivizing users for acquiring virtual goods at a variety of physical locations termed PokéStops or Gyms. There was also excitement among medical and public health communities for the potential use of this innovative and engaging tool to promote regular physical activity. However, racial and ethnic minority groups in low-income, urban areas across the United States soon took notice of the lack of PokéStops within their neighborhoods. This issue was heavily played out on social media under the hashtag #mypokehood [3,4] and inspired researchers to probe the issue further. It was indeed found that neighborhoods consisting of predominantly African American and Hispanic residents in major cities such as Chicago, Detroit, and New York had significantly fewer PokéStops than white and Asian neighborhoods [5,6]. Digital redlining, or limiting a particular community from essential services based on race and ethnicity, was deemed the culprit. The Pokémon GO app developers relied on maps from one of their prior apps that were crowdsourced from a majority white male demographic in commercial areas. This unearthed structural-digital inequity demonstrates how technologies, although not necessarily deliberate, can place certain groups at a *home-court* disadvantage. Community engagement in the development and tailoring of this technology could have thwarted this unfortunate faux pas.

Another unsettling discovery of inequities related to digital innovation is recent reports that smartwatches and other physical activity trackers demonstrate less reliability in accurately monitoring heart rates in people of color, particularly those with darker skin tones [7]. Although there has been scant media attention surrounding this issue, it is well documented in the scientific literature that the inherent optical sensors or *green lights* of these devices are readily absorbed by melanin (skin pigment), presenting a problematic challenge to accurate monitoring of heart rate [7-10]. There are other available technologies to potentially overcome this issue, such as balancing with the use of red light sensors (referred to as near infrared spectroscopy) [7,11]; however, nearly all large manufacturers of these devices rely solely on green light sensors through a process called photoplethysmography as they are simpler and less expensive [11]. One study provided evidence that these devices were *within acceptable error range*, but this existing bias is unacceptable considering the surge in clinical research studies integrating these wearable technologies [9]. This not only limits the potential clinical implications of the use of these devices but could also lead to downstream health disparities. Again, purposeful examination of racial and ethnic differences in the utility of these devices could have been achieved through active community engagement within diverse populations.

## The Divided Digital Revolution

With each hour of the day, hundreds of novel health informatics strategies, telemedicine devices, wearables, and other digital technologies are released at lightning speeds, which has amplified into a US $80 billion industry with a projected increase to over US $500 billion by 2020 [12,13]. Mobile health (mHealth) is a growing field, revolutionizing health promotion and health care delivery through sophisticated digital technologies (eg, mobile/digital apps, SMS/text messaging, and wearable devices), and it provides an unprecedented opportunity to reach and engage communities [14-17]. Racial and ethnic minorities outnumber their white counterparts in the use of mobile/digital apps and are more likely to use their smartphones to access health information [18,19]. African Americans have similar smartphone ownership to the general population (80% vs 81%, respectively) [20], and they are receptive to participating in mHealth research [21]. However, there is a dearth of socioculturally tailored mHealth interventions that include racially and ethnically diverse patients or community members in their development and implementation beyond usability studies [22]. As a result, these acontextually developed innovations may largely benefit health outcomes in one sector of society while inadvertently creating, sustaining, or increasing health disparities in another. This can further perpetuate health inequities through the creation of a new configuration of the digital divide—a paucity of culturally informed or culturally useful health informatics or digital health interventions.

Interestingly, community members have had keen foresight of this potential dilemma and have advocated for more inclusive development processes of these interventions. Community engagement is an evidence-based and practical means to bring overlooked communities into the fold of our rapidly changing health care landscape abound with proliferating digital health innovations. In this viewpoint, we present 2 case studies of community-based mHealth interventions designed and developed alongside community members to effectively address health disparities. Both interventions were born out of community members’ requests for cutting-edge technological interventions to apply within their respective communities and sensible inquiries of “why not us?” to academic researchers. Next, we discuss the origins of health disparities that are essential to understand before engaging with underserved communities. Finally, we present the health informatics and digital health fields with best practices for community engagement in digital innovation.

## Innovation Through Community-Engaged Research: Case Studies

At the heart of community-engaged research is an academic-community partnership for coproducing research to enact social justice for disadvantaged communities through improved health and receipt of high-quality care [23]. In this construct, investigator-driven research is replaced with collaboration, shared decision making, reciprocal relationships, colearning, trust, and transparency [23]. Under the umbrella of community-engaged research is community-based participatory research (CBPR), which incorporates community member input at every phase of the research process, ranging from conception to results dissemination [24,25]. Community members view CBPR as a transformation of traditional research tactics, in which participants may feel *used* and at the mercy of a researcher, to a more active opportunity to work with researchers as equal partners in contouring interventions for the betterment of the health of their communities. There is a gap in the literature of research applying CBPR principles to develop context-sensitive, mHealth innovations that address health inequities [26]. The 2 mHealth interventions described below strategically merged CBPR with health services research for intervention development and implementation (see Table 1).

**Table 1:** Embedded community-based participatory research principles in case studies.

CBPR^a^ principle	FAITH!^b^	PeerTECH^c^
Community as a unit of identity	African American adults affiliated with local African American churches. Shared sociocultural influences of the African American faith community and marginalization in underresourced areas in small and large metropolitan areas in Minnesota.	Older adults, aged 60 years and older, with an SMI^d^. Shared experiences of marginalization and mental health condition with certified peer specialists.
Strengths and resources within the community	Community partner with a long-standing role as a community activist and community outreach director of a federally qualified health center. Established social infrastructure of the Black church for recruitment. Church pastors and church-designated champions, FAITH! Partners leveraged for trust building and *buy-in*.	Certified peer specialists who provide peer support mental health services and are trusted leaders of community. Certified peer specialists who facilitate trust building, co-ownership, and an equitable partnership.
Collaborative partnerships in all research phases	Community members involved in the selection a of mixed methods study design to incorporate community members in intervention development (selected mobile app modality). FAITH! Partners designated to refine recruitment, implementation, and results dissemination processes. Academic partner assisted with capacity-building of health ministries within partnering churches.	Community members involved in weekly research team meetings assisted with the development of the smartphone app content, selection of study site, instruments, hiring, training, and retaining interventionists.
Integrate research results for mutual benefit	Research directly led to an mHealth^e^ intervention aimed at addressing cardiovascular health disparities identified by the academic-community partners within the African American faith community.	Research directly led to a peer-delivered and technology-supported mHealth intervention aimed at addressing early mortality in people with an SMI; health issue identified by academic and community partners.
Cyclical and iterative process	Community members were heavily involved in formative process for mHealth intervention design through iterative focus groups and community meetings.	Community members were actively involved in all research phases from conception and research design to dissemination. Community members are currently involved in the next iteration of PeerTECH.
Colearning and empowerment, with awareness of social inequalities	CSC^f^ established to guide academic partners in project focus and community centeredness. Academic-community partners attend a longitudinal CBPR course to ensure ongoing adherence to principles.	Academic partners learned of the history, philosophy, and practice of certified peer support specialists. Certified peer support specialists learned experientially about conducting and disseminating scientific research.
Incorporate positive and ecological perspectives	Research question and study design were born out of community input and preferences to overcome marginalization. The intervention incorporated relevant psychosocial and sociocultural influences on cardiovascular health (eg, cultural norms of collectivity and spiritual messaging).	Smartphone app was developed based on community input and incorporates a sociocultural and environmental approach to addressing early mortality in people with an SMI, including relationship building skills, stress management, and how to navigate the health care system.
Disseminates knowledge to all partners	Presentations were held in both academic and community settings to share research results. Promoted a culture of health in community dissemination efforts as identified by community partners and CSC (eg, community walk and health fair). Culturally tailored infographics were developed to highlight results for participants, CSC, and public. Academic-community partners coauthored manuscripts/grants and copresented at scientific and community meetings.	Presentations were held in both academic and community settings (international, national and regional conferences) to share research results. Blogs were written by patient partners and certified peer specialists. Academic-community partners coauthored peer-reviewed manuscripts/grants and copresented at scientific meetings.

^a^CBPR: community-based participatory research.

^b^FAITH!: Fostering African-American Improvement in Total Health.

^c^PeerTECH: Peer- and Technology-Supported Self-Management Training.

^d^SMI: serious mental illness.

^e^mHealth: mobile health.

^f^CSC: community steering committee.

## Fostering African-American Improvement in Total Health

The Fostering African-American Improvement in Total Health (FAITH!) intervention, cultivated from a community desire to improve the cardiovascular health of the African American community, is a translation of a face-to-face, church-based health education program into an mHealth intervention [14,27]. Community members expressed directly to the collaborating research team the dire need for easily accessible and trusted health information and an infrastructure for social support through the use of mobile technology [28]. This was further fueled by their mutual desire with the project leader to confront persistent cardiovascular disease (CVD) disparities in Minnesota, as African Americans have a higher CVD incidence and nearly double the CVD mortality rate than whites [29]. Prior efforts to mitigate these disparities have been hindered by social marginalization and structural racism [30].

**Figure 1:**
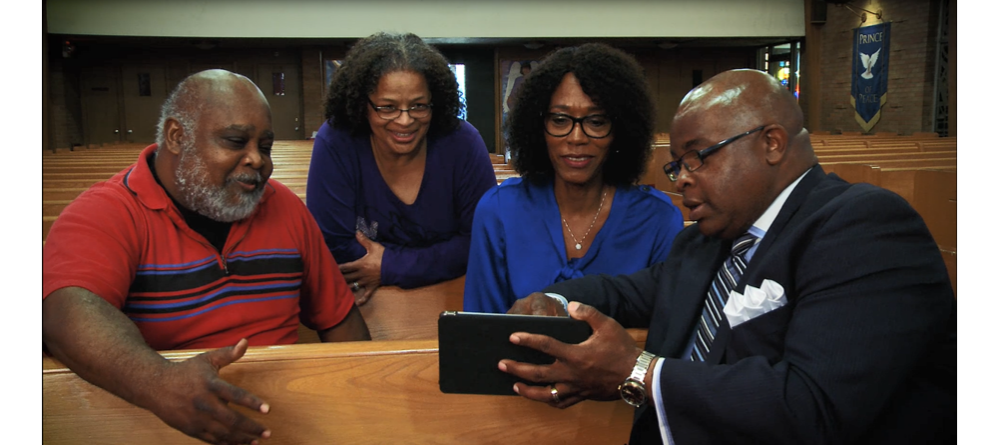
Community members interacting with a community-based mobile health intervention, the *FAITH!* (Fostering African-American Improvement in Total Health) *App*. Researchers and African American churches partnered to codevelop the *FAITH! App* to promote cardiovascular health within faith communities. Used with permission from the Mayo Foundation for Medical Education and Research.

FAITH! Partners, or church-designated champions, were intricately involved in defining the research questions and study design to assess the feasibility of the mHealth intervention and in selecting its actual delivery modality in the form of a mobile app. An iterative, formative design process was employed to jointly create the *FAITH! App* with African American community partners and an interdisciplinary research team including clinicians, technologists, and social and behavioral scientists [31]. FAITH! Partners provided the research team with valuable insights on the psychosocial needs and preferences of the African American faith community, which undoubtedly improved the design of the intervention. For example, incorporation of biblical scriptures and spiritual messaging was a strong recommendation from the community partners during the app prototyping phase. Our inclusion of these inferences was viewed as cultural humility by the research team and resulted in the high ratings of the app’s acceptability, usability, and satisfaction by our study participants (see Figure 1) [31].

**Figure 2:**
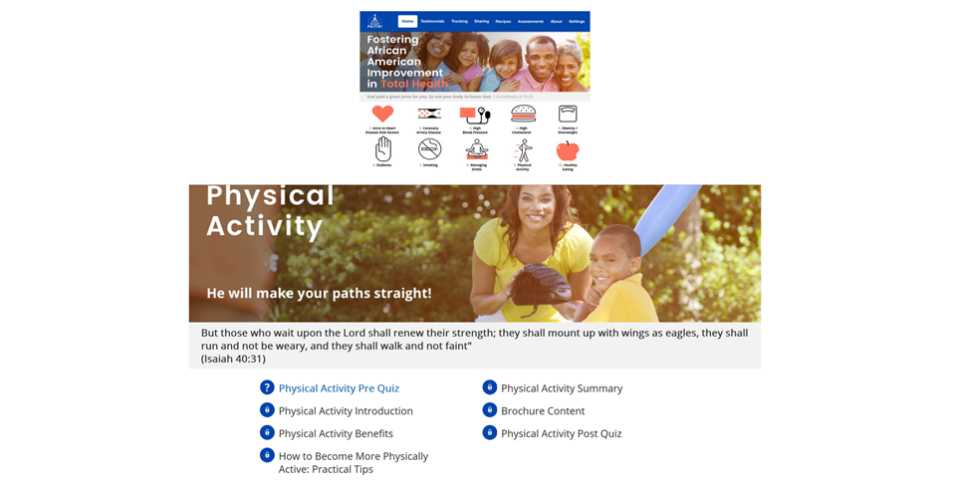
Screenshots of the co-designed *FAITH!* (Fostering African-American Improvement in Total Health) *App* home page and education module. Used with permission of the Mayo Foundation for Medical Education and Research.

Further integration of evidence-based behavioral change theoretical frameworks into the app features resulted in a culturally aligned intervention that positively impacted the cardiovascular health of the study participants (blood pressure, diet, and physical activity; all *P* values<.04) [32]. See Figure 2 for images of the app homepage and sample education module.

Unsurprisingly, community involvement and trust building facilitated exceptional recruitment and retention rates of study participants (100% and 98%, respectively) not traditionally involved in the research process [14,32]. Culturally tailored, visual depictions of study results through infographics were developed with community partner feedback and were distributed within the partnering churches, at local community events, and at public health departments. The academic-community partnership has recently secured federal funding to expand the reach of the FAITH! intervention. On the basis of community input, there are also ongoing plans to disseminate the FAITH! App not only to the African American faith community at large but also within community health centers [33]. In addition, the genuine relationships forged between the academic medical institution and the marginalized community in codeveloping the intervention strengthened the diversity and inclusion efforts led by the institution, including revitalizing its branding strategy for patient accessibility [34].

## Peer- and Technology-Supported Self-Management Training

**Figure 3:**
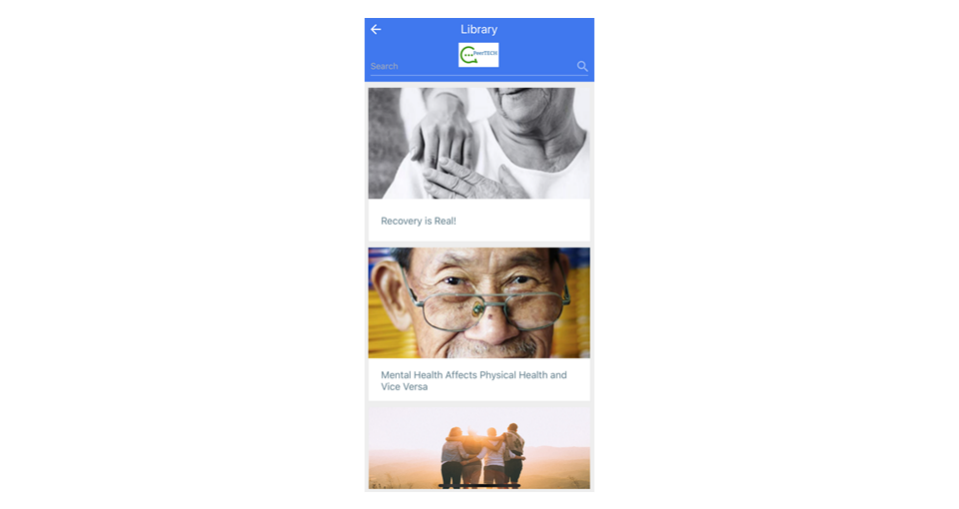
Screenshot of the educational library in the co-designed PeerTECH (Peer- and Technology-Supported Self-Management Training) app.

Another CBPR partnership focused on addressing premature mortality in people with a serious mental illness (SMI; eg, bipolar disorder, major depressive disorder, and schizophrenia), which was identified by the partnering academic-community team as a major health disparity affecting this vulnerable population [35]. This partnership led to the cocreation of a smartphone app–based intervention, *Peer- and Technology-Supported Self-Management Training* (PeerTECH), which is aimed at the *simultaneous* management of mental and chronic health conditions in patients aged 60 years and older (see Figure 3) [23,36].

PeerTECH was developed in *equal* partnership between patients, certified peer specialist (CPS) leaders, and scientists from idea conception, defining of research questions, intervention development, and usability testing extending to dissemination. CPS leaders are individuals with a lived experience of a mental health condition who are trained to provide peer support in mental health services. They are deep-rooted advocates for developing programs with community input and the resounding motto of “nothing for us, without us.” CPS leaders expressed concern to the research team over the lack of resources available to engage older adults living with SMI and wanted to devise a solution to improve outreach to these individuals who otherwise might not engage in traditional mental health services [37]. Digitally supporting geriatric mental health was identified as an innovative means to overcome geographic barriers by remotely delivering customized services based on patients’ preferences and recovery goals while simultaneously addressing comorbidities [38,39]. An iterative app coproduction process with CPS input transformed the app from a highly medicalized self-management approach to one with an emphasis on recovery through a self-management app. For instance, instead of solely targeting psychiatric symptoms from a medical standpoint, PeerTECH utilizes a biopsychosocial approach and targets multiple dimensions of health including, but not limited to, *how to make friends* (social support), *what to do when you are lonely* (loneliness), and *how to stick up for yourself at the doctor’s office* (self-advocacy). By including the insights of older adult patients with an SMI, PeerTECH has the potential to promote widescale acceptability among this highly marginalized group and improve population health.

PeerTECH was delivered in person by a CPS, who has the personal experience of an SMI and skills to provide services to a patient with similar health issues. PeerTECH was further augmented with the smartphone app [40]. PeerTECH sought to improve psychiatric and chronic disease management among patients with an SMI through self-monitoring of psychiatric distress, medication adherence, and peer support. PeerTECH was found to be feasible and acceptable among patients and CPS leaders [41]. The use of PeerTECH was associated with statistically significant improvements in psychiatric self-management (*P*<.001) and improvements in medical self-management, hope, quality of life, and empowerment. This coproduction team has presented to international audiences and has received foundation and federal funding to continue their work.

Both of the case study projects confront health disparities through digital health interventions at multiple levels (individual and meso levels) by tapping into existing social and community networks [42]. Both interventions support marginalized populations through collective mobilization and enhancement of resources, reduction of social isolation through social networking, and sharing of knowledge through technology-mediated solutions to promote positive health behaviors. By *leveling up* to upstream contextual and societal influences on health and health disparities [43], these interventions provide comprehensive yet pragmatic models for future health informatics and digital health design and implementation. We hope that these exemplars of integration of user-centered design (UCD) and participatory design (PD) processes into technology development can serve as examples for others as we usher in the accelerated advancement of the health informatics and digital health fields.

## Understanding the Origins of Health Disparities and the Social Determinants of Health

Before engaging with communities, researchers, clinicians, and regulatory agencies focused on health informatics and digital health must first understand and critically examine the origins of health disparities among marginalized and underresourced communities. A lack of acknowledgment of these inequities could lead to their further propagation and widen the digital divide by disproportionately providing beneficial technologies to nonmarginalized groups that already have health-related advantages [42,44].

Health disparities are metrics to monitor advancement toward health equity [45]. According to the World Health Organization, *health equity* implies that everyone should have a fair opportunity to attain their full health potential and that no one should be disadvantaged from achieving this potential [46]. Unfortunately, the reality is that many are not afforded this *golden* opportunity to achieve optimal health as a result of complex socioeconomic, political, environmental, and sociological factors [24]. These psychosocial factors or social determinants of health (SDOH) are critical in predicting health outcomes and are tied to the majority of health inequities [47-49]. They are defined as “conditions in which people are born, grow, live, work and age” and are “shaped by the distribution of money, power and resources at global, national and local levels” [47].

The SDOH do not occur at random but cluster at the intersectionality of social identities/position (such as one’s race or ethnicity, gender, or educational attainment), which may have a multiplicative, adverse impact on health outcomes [50-55]. Racial and ethnic minority populations are faced with a unique milieu of disenfranchising SDOH, which include inadequate access to quality health care and health care providers, multitiered and systemic racism, food and housing insecurity, and lack of employment opportunities, which further impede their opportunities for ideal health and wellness. Despite progress made toward improving the health of the US population as a whole, racial and ethnic minorities shoulder the heaviest burden of health disparities related to higher prevalence and premature mortality from chronic health conditions [56,57] including CVD [58-60], diabetes [61,62], obesity [63], and SMI [64,65]. There is also evidence linking stress related to racial discrimination to increased risk of these chronic health conditions and negative health outcomes [66-71]. In addition, reduced community-level social capital stemming from oppression/privilege and institutional/structural racism toward African Americans has been correlated with a hindrance of their economic prosperity and increased mortality risk [55,72]. Moreover, racial and ethnic minority groups have been historically faced with negative stereotyping bias [73-76] and unjust criminal sentences [77,78], which unfortunately have been recently promulgated through present-day data discrimination via search engines [79] and machine learning algorithms [80,81]. An intersectional approach considers the complex interaction of all these factors and could synergistically address the strata of the SDOH in tandem with health disparities. This approach is not *one size fits all*, but it proactively aims to design multiaxis interventions and programmatic strategies to meet the unique needs of vulnerable populations.

Addressing the SDOH through innovative digital technologies is a promising channel to overcome health inequities experienced by racial and ethnic minorities and other underresourced populations, including older adults, rural residents, and the economically disadvantaged. This will require innovators to demolish insulated siloes and reach beyond the confines of traditional research and clinical settings to better understand underserved communities. This approach is also known as sociotechnical, or one with a recognition of the interrelatedness of the *social* and *technical* factors of a particular environment to create optimal conditions or tools to maximize productivity and well-being [26,82]. Leaders within the Computing Community Consortium and the Society for Behavioral Medicine from interdisciplinary fields, including computing, health informatics, behavioral medicine, and health disparities, have recently called for an integrative research agenda to improve the health of the socioeconomically disadvantaged through advanced sociotechnical interventions [43,83]. As a consensus, the group agreed that reducing disparities will require a method that engages the affected populations “at all stages of intervention design, implementation and evaluation.” This approach moves beyond an instrumental view of the technical and logistical aspects of interventions to that of engendering value in the sociocultural-centric context of the *where and how* of intervention deployment for exceptional patient experience. Ultimately, this will allow for the development of multidimensional health informatics and digital health interventions with a more informed awareness of the social context in which people actually live, learn, work, play, and pray.

## The Roles of Health Informatics and Digital Health in Advancing Health Equity

### Community Engagement for Digital Intervention Design

We understand that an all-encompassing CBPR co-design approach between researchers and end users in the design of digital interventions (as integrated in the case studies above) may seem overwhelming. CBPR can also lend challenges in academics for a multitude of reasons, including the pressures of scholarly productivity mainly driven by timeline constraints, resources, lack of expertise, and funding. However, there are several other sociotechnical approaches along the spectrum of community engagement that scientists and innovators may adopt for technology design and implementation. These include UCD [84,85] and PD [86,87], which also align with the overarching goal of CBPR to incorporate preferences and perspectives of intended end users into technology design. These approaches also allow for the integration of similar methodologies of CBPR adaptable to varying degrees of community involvement (ie, focus groups and think-aloud sessions). Nonetheless, these design strategies can be applied to develop technology interventions within the social construct lens of diverse communities rather than solely based on developer-driven needs. Interventions designed with community involvement are better equipped to address the inequities that their contexts create—which is especially important for racial and ethnic minority groups.

The UCD approach has a central theme of involvement of end users throughout the technology development process to optimize value and usability (inclusive of safety, efficiency, and effectiveness) for users [88]. Although previously considered costly and time consuming, UCD actually reduces development time considerably by integrating real-time quality improvement and prototype testing with users [89]. This not only improves functionality but also increases the probability that interventions will promote positive health behaviors or outcomes and that intended users will embrace and sustain use of the technology [90]. An example of how UCD can yield technological interventions tightly coupled to specific user needs and challenges to address the SDOH is that of an initiative to alleviate transportation barriers to medical appointments for underserved patients at Hennepin County Medical Center in Minneapolis, Minnesota [91]. On the basis of the bidirectional exchange between researchers with patients and health care providers, an electronic health record (EHR)–linked SMS text message system was developed to provide patients with free, convenient transportation via rideshare services to and from outpatient clinics. This is a win-win situation for patients and health care providers as it improves health care access and helps in mitigating unnecessary emergency department visits and hospitalizations. Similar to UCD, PD harnesses the collaboration of end users with researchers and developers in iterative co-design cycles to increase intervention acceptability and engagement of target audiences [92,93]. PD methodologies have been particularly successful in the development of patient-centered digital interventions to stigmatized populations, including those to deliver language services for individuals with limited English proficiency [94], mental health services to low-income women in urban areas [95], social support for people living with HIV [96], and research participation outlets to underrepresented minority groups [97,98]. Both UCD and PD have the advantages of reducing the lag between research and development to translation, which is vital given the rapid pace of technology turnover. In addition, these approaches offer continuous insight into the dynamic nature of individuals’ environment, which can prevent outdated/stagnant interventions with limited value and lead to the discovery of modernized solutions over time.

## Community Engagement for Epidemiologic Surveillance and Population Health Informatics

Reliance on data and analytics to identify and surveil epidemics and allocate resources to protect the health of underserved populations was traditionally the foundation and moral fiber of medicine and public health [99]. In fact, the health informatics field itself was spurred by the Centers for Medicare and Medicaid Services *meaningful use* incentive program, which encouraged widespread health system adoption of the EHR to optimize patient care and health outcomes [41,100]. The *timely intelligence* of the rapidly evolving digital age presents an inviting and yet germane doorway to leverage robust data and technology in ways unimaginable to address health disparities and upstream SDOH [99].

However, it is important to recognize that not all communities have readily available access to high-quality population data or even the capacity for data collection, sharing, or analysis to identify or monitor health inequities among racial and ethnic minority or marginalized populations [101]. For example, local health departments in rural settings are faced with a *double disparity* as they are ill-equipped for data-informed decision making to combat health disparities, which negatively influences relationships with community organizations. Overcoming this *information system challenge* through improved informatics infrastructure could advance community-engaged approaches to utilizing population-level data to understand and act upon health issues faced by underserved communities. Community health informatics, a subdomain of health informatics, aims to generate and maintain relevant data on community health needs assessments from community-level stakeholders [102]. Partnering with community members in gathering and synthesizing *granular and place-based* data (eg, from churches, barbershops, tribal areas, or community meetings) could promote health equity through culturally appropriate solutions to ascertained health disparities.

In addition, population health informatics tools, including EHRs, could be tapped as knowledge hubs (or repositories) by health care ecosystems and public health agencies to disentangle the determinants aggravating health disparities affecting socially disadvantaged groups [99]. Synthesizing data from these hubs could better detect, track, respond to, and predict sources of health disparities such as differential, guideline-concordant preventive screening and care; poor patient-provider communication from stereotyping and bias; and errors in clinical decision making, which all drive poor health outcomes among racial and ethnic minorities and other vulnerable populations. It has been postulated that EHR data streams could potentially facilitate the creation and longitudinal surveillance of standardized quality metrics of health equity for use by health systems to reduce disparities [103,104], lower health care costs, and ultimately improve patient experiences and health outcomes. EHRs also provide an extraordinary platform for enhanced ascertainment and documentation of the SDOH, which could lead to improved care coordination, patient-clinician shared decision making, and resource allocation to underserved patients [48,105]. Pairing EHR monitoring technologies and sophisticated data platforms with interdisciplinary service providers (community health workers, nurses, and social workers) within resource-constrained settings could address the SDOH through population health management [106-108].

There is also a need for more culturally and linguistically sensitive strategies to increase access, uptake, and engagement with patient portal EHRs by racial and ethnic minorities while accommodating varying levels of electronic health and health literacy as well as technology experience and privacy concerns [22,109-112]. A failure to do so could widen the digital divide by disallowing these patients the opportunity to benefit from this form of health care access. Likewise, we must also recognize that machine learning algorithms are oftentimes developed from racially homogenous data flawed with intrinsic biases [81]. We must not let our enthusiasm about the glowing promise of these *superhuman* models blind our view of the potential health inequities that they could propagate among vulnerable populations through inaccurate predictions or withholding of resources [81]. Deepening our understanding of the role of high-quality EHR and fairness in machine learning in addressing health disparities through rigorous research could inform the design of novel technologies (including artificial intelligence) at the individual, health care provider, health systems, and community levels to promote health equity. Embedding the unique perspectives of patients and community members into the development of these technologies and advanced computing power has transformative potential to reach this goal.

Textbox 1 provides further recommendations for best practices in strategic design and implementation of health informatics and digital health interventions in marginalized communities. These recommendations are also provided in the context of the enlightening experiences with the design, implementation, and translation of FAITH! and PeerTECH. These recommendations altogether are indispensable in capacity building for both the research team and the community in tackling and preventing health disparities. Interventions created with these practices in mind will increase the likelihood of their success in informing and shaping further digitally supported interventions, health promotion strategies, digitally supported health systems, health systems informatics tools, and health care policies for the benefit of all populations.

**Best practices in strategic design and implementation of health informatics and digital health interventions in marginalized communities.**

1. Increase recruitment and retention of diverse populations throughout the research and development process to allow for assessment of differential responses/outcomes of technologies and to mitigate preferential access to certain population sectors.
2. Leverage established stakeholders and trusted social networks to understand the strengths and resources within underserved communities.
3. Understand the social context of potential end users and populations as this allows for understanding of the social determinants of health and how these are embedded within systems of inequality within underserved communities.
4. Integrate community engagement through user-centered design or participatory design to better understand potential end users’ needs and preferences to develop culturally relevant and meaningful interventions.
5. Gain an understanding of community partner technology infrastructure for capacity building to support and strengthen community-based health informatics interventions.
6. Plan the appropriate amount of time and resources to devote to community engagement processes for intervention development and sustainability.

## Conclusions

In the current irresolute climate of national health care reform, it is essential for researchers, public health practitioners, informaticians, and technologists working in health informatics and digital health to embrace implementation science and community engagement in our collective quest to eliminate health disparities. With the exponential growth of these fields, we must ensure their *meaningful use* of applications for the betterment of the health of marginalized and underserved communities. Innovation through community engagement presents opportunities to bolster technological advancements to intercept health inequities.

Everyone benefits when community members are fully vested and included in intervention development and implementation. Their valuable perspectives toward addressing population health within the context of their social and physical environments lead to more successful interventions. Investigators must not only *think outside the box* but also examine the box itself and its surroundings to attain real, lasting change to impact health disparities within our communities. This intentional decision to *meet people where they are* in the community, whether culturally or digitally, is a return to the medical profession’s core principles of altruism and benevolence and a journey *back to the future* to achieve health equity for all.

## Abbreviations

CBPR: community-based participatory research
CPS: certified peer specialist
CVD: cardiovascular disease
EHR: electronic health record
FAITH!: Fostering African-American Improvement in Total Health
mHealth: mobile health
NIH: National Institutes of Health
PD: participatory design
PeerTECH: Peer- and Technology-Supported Self-Management Training
SDOH: social determinants of health
SMI: serious mental illness
UCD: user-centered design
